# Repetitive Treatment with Diluted Bee Venom Attenuates the Induction of Below-Level Neuropathic Pain Behaviors in a Rat Spinal Cord Injury Model

**DOI:** 10.3390/toxins7072571

**Published:** 2015-07-10

**Authors:** Suk-Yun Kang, Dae-Hyun Roh, Jung-Wan Choi, Yeonhee Ryu, Jang-Hern Lee

**Affiliations:** 1 KM Fundamental Research Division, Korea Institute of Oriental Medicine, Daejeon 305-811, Korea; E-Mails: sy8974@kiom.re.kr (S.-Y.K.); cjw214@kiom.re.kr (J.-W.C.); 2 Department of Oral Physiology and Research Center for Tooth and Periodontal Tissue Regeneration, School of Dentistry, Kyung Hee University, Seoul 130-701, Korea; E-Mail: dhroh@khu.ac.kr; 3 Department of Veterinary Physiology, College of Veterinary Medicine and BK21 Program for Veterinary Science, Seoul National University, Seoul 151-742, Korea

**Keywords:** bee venom, spinal cord injury, mechanical allodynia, thermal hyperalgesia, glia, acupuncture

## Abstract

The administration of diluted bee venom (DBV) into an acupuncture point has been utilized traditionally in Eastern medicine to treat chronic pain. We demonstrated previously that DBV has a potent anti-nociceptive efficacy in several rodent pain models. The present study was designed to examine the potential anti-nociceptive effect of repetitive DBV treatment in the development of below-level neuropathic pain in spinal cord injury (SCI) rats. DBV was applied into the Joksamli acupoint during the induction and maintenance phase following thoracic 13 (T13) spinal hemisection. We examined the effect of repetitive DBV stimulation on SCI-induced bilateral pain behaviors, glia expression and motor function recovery. Repetitive DBV stimulation during the induction period, but not the maintenance, suppressed pain behavior in the ipsilateral hind paw. Moreover, SCI-induced increase in spinal glia expression was also suppressed by repetitive DBV treatment in the ipsilateral dorsal spinal cord. Finally, DBV injection facilitated motor function recovery as indicated by the Basso–Beattie–Bresnahan rating score. These results indicate that the repetitive application of DBV during the induction phase not only decreased neuropathic pain behavior and glia expression, but also enhanced locomotor functional recovery after SCI. This study suggests that DBV acupuncture can be a potential clinical therapy for SCI management.

## 1. Introduction

One of the pain therapies is the use of chemical stimulation into an acupuncture point to produce an analgesic effect and to reduce pain severity. In this regard, the injection of diluted bee venom (DBV) into an acupuncture point, termed apipuncture, has been used clinically in traditional Korean medicine to produce a significant analgesic effect in human patients [1,2]. Many experimental studies have demonstrated that injecting DBV into the Joksamli (ST36) acupuncture point produces a robust anti-nociceptive effect in various pain animal models, such as the writhing test, the formalin test, the carrageenan-induced inflammatory pain test and arthritis models [3,4,5,6]. Furthermore, we demonstrated that this DBV-induced anti-nociceptive effect is associated with the activation of descending coeruleospinal noradrenergic pathways, which subsequently activate spinal alpha-2 adrenoceptors [3,7,8]. DBV stimulation of ST36 also inhibits the activation of spinal astrocytes in a mouse formalin test [3]. In particular, we showed that a single injection of DBV (0.25 mg/kg) into ST36 temporarily alleviated thermal hyperalgesia [9] and that repetitive stimulation using DBV significantly alleviated neuropathic pain-induced mechanical and cold allodynia in a sciatic nerve chronic constrictive injury (CCI) model of rats [7,8]. However, the precise roles of repetitive DBV treatment in the induction and maintenance phases of central neuropathic pain have not been examined. 

Spinal cord injury (SCI), which is caused by direct traumatic damage to the spinal cord, has been related to many clinical complications, including functional disability, urinary tract problems, autonomic dysreflexia, altered sensations and pain [10,11,12]. These patients often have experiences of several types of pain; central chronic pain syndrome, which exhibits mechanical allodynia and thermal hyperalgesia, is one of the most common causes for a reduced quality of life [13,14]. Especially, below-level pain after SCI represents a clinically-significant symptom of central neuropathic pain that is very difficult to treat effectively [15,16]. Several experimental models of SCI have been developed to determine the detailed mechanisms and therapeutic strategies to treat SCI. The most widely-used models are rat SCI contusion, excitotoxic and hemisection models [14,17,18,19]. In this study, the rat SCI hemisection model was chosen, because this model is widely used to verify the mechanism behind SCI-induced chronic pain development. 

Recently, a number of studies have reported the potential role of spinal astrocytes and microglia in both postoperative pain and neuropathic pain [20,21,22,23,24]. Moreover, intrathecal treatment with glia inhibitors, such as minocycline (a microglia inhibitor) and propentofylline (a glia modulating agent), reduced below-level neuropathic pain behaviors in SCI rats [25,26,27]. However, although there is some evidence that glial cells are activated during the development of SCI-induced neuropathic pain, the precise mechanisms underlying glial activation, particularly in lumbar segments distant from the spinal cord injury site, are poorly understood. 

Based on the above-mentioned studies, we hypothesized that repetitive DBV treatment into an acupoint reduces SCI-induced mechanical allodynia and thermal hyperalgesia and that this reduction is mediated by the suppression of spinal astrocyte or microglia activation. Thus, the present study was designed to examine the following: (1) whether repetitive DBV acupuncture point treatment for five days during the induction and maintenance phases following thoracic 13 (T13) spinal cord hemisection would produce a more potent and prolonged analgesic effect compared to controls that received repetitive injections of vehicle; (2) whether the anti-nociceptive effect of DBV is mediated by the modulation of spinal astrocyte and microglia activation; and (3) whether repetitive DBV treatment affects motor functional recovery in SCI rats.

## 2. Results

### 2.1. Effect of Repetitive DBV Treatment during the Maintenance Phase of Spinal Cord Injury-Induced Pain

**Figure 1:**
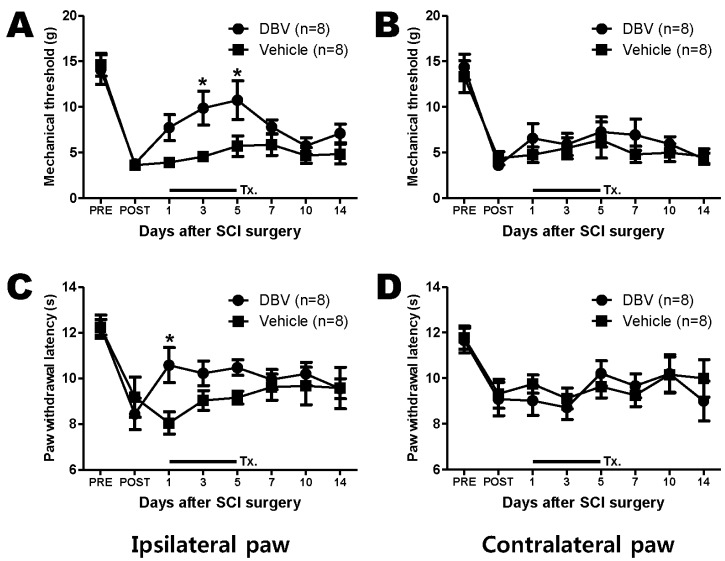
Graphs illustrating the effects of repetitive diluted bee venom (DBV) or vehicle on the maintenance phase of mechanical allodynia (**A**,**B**) and thermal hyperalgesia (**C**,**D**) in spinal cord injury animals. (**A**) Repetitive daily treatment with DBV (from 15 to 20 days post-surgery, twice a day) increased the paw withdrawal threshold by mechanical stimuli for the period of DBV treatment in the ipsilateral hind paw (* *p* < 0.05 compared to the vehicle-treated group); (**B**) whereas the contralateral paw did not display any differences compared to the vehicle-treated group; (**C**) repetitive DBV treatment reversed the spinal cord injury (SCI)-induced decrease in the paw withdrawal latency (s) to noxious thermal stimuli compared to the vehicle-treated group (* *p* < 0.05); (**D**) no significant difference in the paw withdrawal latency was observed in the contralateral paw between the DBV and vehicle-treated groups. Two-way ANOVA followed by Bonferroni’s test. PRE; one day before SCI surgery, POST; 15 days after SCI surgery. Tx.: DBV or vehicle treatment.

Spinal cord hemisection produced prominent mechanical allodynia and thermal hyperalgesia, as shown in Figure 1. During the maintenance phase, repetitive DBV treatment was administered twice a day from 15 to 20 days post-surgery. Repetitive DBV treatment significantly increased the decrease in the paw withdrawal threshold in the ipsilateral hind paw by SCI surgery at three and five days after DBV treatment (* *p* < 0.05, compared with saline-treated groups) (Figure 1A); however, mechanical allodynia of the contralateral paw did not show any change compared to saline-treated animals (Figure 1B). 

As shown in Figure 1C, repetitive DBV treatment during the maintenance phase increased paw withdrawal latency to noxious thermal stimulus only Day 1 after DBV treatment compared with the repetitive saline-treated group (* *p* < 0.05). In the contralateral paw, repetitive DBV treatment had no effect on SCI-induced thermal hyperalgesia (Figure 1D). 

### 2.2. Effect of Repetitive DBV Treatment during the Induction Phase of Spinal Cord Injury-Induced Pain

**Figure 2:**
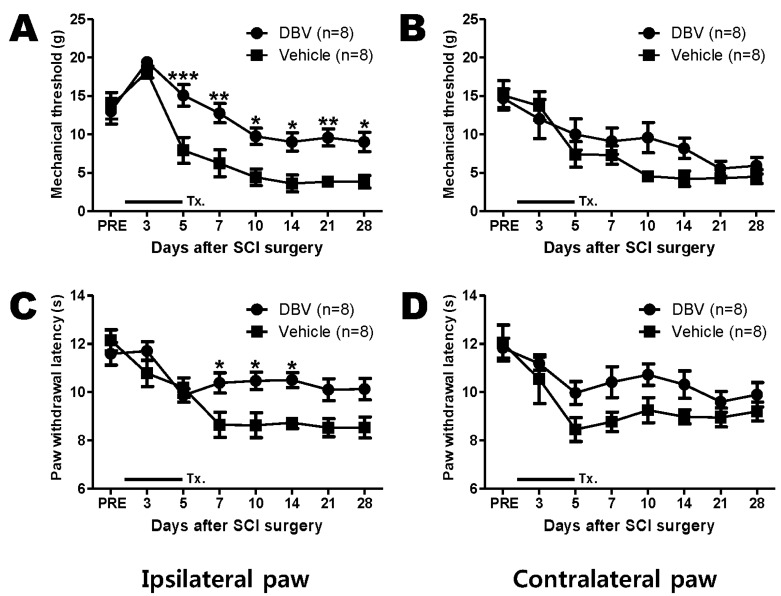
Graphs illustrating the effects of repetitive DBV or vehicle treatment during the induction phase on mechanical allodynia (**A**,**B**) and thermal hyperalgesia (**C**,**D**) in spinal cord injury animals. (**A**) Repetitive daily treatment with DBV (twice a day from one to five days post-surgery) suppressed the induction of SCI-induced mechanical allodynia in the ipsilateral hind paw compared with vehicle-treated rats (* *p* < 0.05, ** *p* < 0.01, *** *p* < 0.001); (**B**) whereas the contralateral paw did not display any differences; (**C**) repetitive treatment with DBV reversed the SCI-induced decrease in the paw withdrawal latency (s) to noxious thermal stimuli compared to the vehicle-treated group (* *p* < 0.05); (**D**) no significant difference in the paw withdrawal latency was observed in the contralateral paw between the DBV- and vehicle-treated groups. Two-way ANOVA followed by Bonferroni’s test. PRE: one day before SCI surgery; Tx.: DBV or vehicle treatment.

The withdrawal response threshold to innocuous mechanical stimuli and withdrawal response latency to noxious thermal stimuli were measured in repetitive DBV and vehicle treatment groups during the induction phase (twice a day from one to five days post-surgery, Figure 2). Groups treated with saline (vehicle, *n* = 8) in the ipsilateral paw showed an approximately 6 to 7-g threshold for mechanical allodynic behaviors in both paws by five days post-surgery. The peak was reached at Day 14 (10 days after the termination of injection). Repetitive DBV-treated groups (DBV, *n* = 8) displayed potently suppressed pain induction in the ipsilateral paw as early as five days post-surgery (* *p* < 0.05, ** *p* < 0.01 and *** *p* < 0.001, compared with saline-treated groups; Figure 2A). In the contralateral paw, DBV-treated groups did not display significantly altered SCI-induced mechanical allodynia compared to the saline-treated control group (Figure 2B). Repetitive DBV injection for five consecutive days during the induction phase significantly increased the SCI-induced decrease in the paw withdrawal latency to noxious thermal stimulus beginning seven days post-SCI surgery compared with the repetitive saline-treated group (* *p* < 0.05; Figure 2C). In the contralateral paw, groups treated with repetitive DBV for five days showed a tendency toward increasing the paw withdrawal latency after DBV treatment; however, this increase was not significant (Figure 2D).

### 2.3. Effect of Repetitive DBV Treatment during the Induction Period on Glia Expression after Spinal Cord Injury

**Figure 3:**
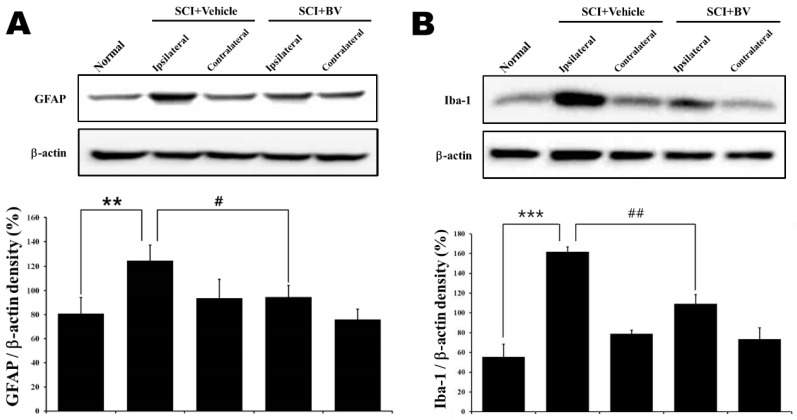
Graphs illustrating the effects of repetitive DBV or vehicle treatment during the induction phase on astrocyte (**A**) and microglia (**B**) expression in the lumbar spinal cord of SCI rats. (A) Western blot data confirmed the effect of repetitive DBV administration on GFAP (astrocyte marker) expression in the spinal cord dorsal horn 14 days after SCI surgery. Vehicle-treated rats displayed significantly increased GFAP expression in the spinal cord compared to normal rats (** *p* < 0.01), and repetitive DBV-treated rats displayed significantly decreased GFAP expression in the spinal cord compared to the vehicle-treated group (# *p* < 0.05). (**B**) In the ipsilateral spinal cord, Iba-1(microglia marker) expression increased following SCI surgery compared to the Iba-1 expression level in normal rats (*** *p* < 0.001), and repetitive DBV-treated rats displayed significantly decreased Iba-1 expression compared to the vehicle-treated group (## *p* < 0.01).

To determine how repetitive DBV treatment might affect glia, astrocyte and microglia expression, the Western blot assay was performed on the lumbar spinal cord dorsal horn at 14 days after SCI surgery. Astrocytes can respond quickly to various pathological stimuli, and this response is related to an increase in GFAP. Repetitive saline injection during the induction phase significantly increased GFAP expression in the spinal dorsal horn compared with that of normal animals (** *p* < 0.01), and repetitive DBV treatment at ST36 suppressed SCI-enhanced GFAP expression (# *p* < 0.05), suggesting that DBV treatment has a potent anti-nociceptive effect on SCI-induced central neuropathic pain. However, the contralateral spinal cord dorsal horn did not reproduce this suppressive effect of DBV (Figure 3A). In Figure 3B, repetitive saline injection also showed a significant increase in Iba-1 expression in the ipsilateral spinal cord dorsal horns compared to that of normal animals (*** *p* < 0.001), and DBV-treated rats displayed significantly decreased Iba-1 expression compared to the vehicle-treated group (## *p* < 0.01).

### 2.4. Effect of Repetitive DBV Treatment on Motor Function Recovery after Spinal Cord Injury

**Figure 4:**
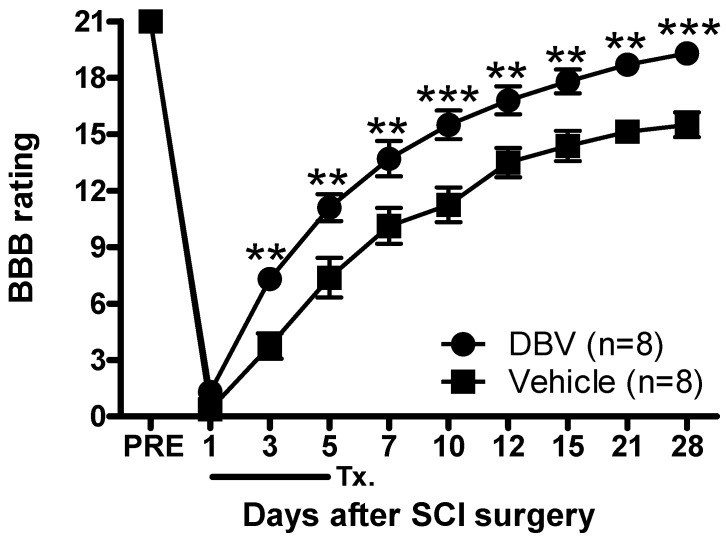
Graphs illustrating the effects of repetitive DBV or vehicle treatment during the induction phase on the recovery of motor function in SCI rats. The Basso, Beattie and Bresnahan (BBB) scores were 21 in all SCI groups before hemisection. Immediately after recovering from anesthesia, hemisected animals appeared to show a loss of ipsilateral hindlimb function as indicated by BBB scores of zero for each group. Animals treated repeatedly with DBV from Days 1 to 5 after hemisection showed faster functional recovery rates than those treated with vehicle (** *p* < 0.01, *** *p* < 0.001). PRE: one day before SCI surgery; Tx.: DBV or vehicle treatment.

Before hemisection, the Basso, Beattie and Bresnahan (BBB) scores were 21 in the repetitive DBV and saline groups (Figure 4). Immediately upon emerging from anesthesia, hemisected animals showed a dramatic loss of ipsilateral hindlimb function as indicated by BBB scores of zero for each group. From Days 1 to 5 after hemisection, rats treated with DBV during the induction phase displayed faster functional recovery rates throughout the four-week period following SCI surgery than those treated with vehicle (** *p* < 0.01 and *** *p* < 0.001 compared with saline-treated groups). 

## 3. Discussion

This study demonstrated that repetitive injections of DBV into the Joksamli acupuncture point during the induction phase (one to five days after SCI) of below-level neuropathic pain significantly produce a more potent and prolonged anti-nociceptive effect compared to repetitive DBV treatment during the maintenance phase (15 to 20 days after SCI) or repetitive injections of the vehicle. Importantly, repetitive treatment with DBV had less of an effect when administered during the maintenance phase. Acupuncture therapy, including manual acupuncture, electro-acupuncture and DBV therapy, produces a gradually increasing anti-nociceptive effect in chronic pain patients when injected repetitively over the course of several days, weeks or months [28]. Huang *et al.* found that high-frequency electro-acupuncture treatment twice a week for four weeks produced a significant reduction in mechanical hyperalgesia by the third and fourth weeks of treatment, whereas it caused no effect on thermal hyperalgesia in a chronic inflammatory pain model of rats [29]. In general, bee venom (BV) contains a number of potential pain-related substances, including melittin, histamine and phospholipase A2, and this mixture of biologically-active substances is able to induce toxic effects, contributing to certain clinical signs or symptoms of envenomation. Human responses to BV include small edema, redness, extensive local swelling, anaphylaxis, systemic toxic reaction and pain [30]. Thus, the use of BV always requires great care.

By contrast, BV has been also used in Oriental and Korean medicine to reduce pain and inflammation. We demonstrated previously that repetitive DBV injection into the Joksamli acupuncture point twice a day for two weeks could significantly decrease mechanical and cold allodynia and thermal hyperalgesia in CCI-induced neuropathic rats [7,8], whereas single DBV injection into the acupuncture point temporarily suppressed thermal hyperalgesia (up to 45 min after DBV injection), but not mechanical allodynia in CCI rats [9]. Our results demonstrate that repetitive injections of DBV into the acupuncture point play an important role during early induction, but not during maintenance of pain behaviors associated with central neuropathic pain conditions. To exclude a possible influence of the temporary anti-nociceptive effect of DBV (observed immediately after each daily DBV injection) on the long-term effects of repetitive DBV treatment, we performed the pain behavioral tests in the afternoon between 6 and 10 h after DBV injection. During the sixth to 10th hour after DBV injection, the temporary anti-nociceptive effect was no longer shown, thus the collected anti-nociceptive data indicate the net long-term effect produced by repetitive DBV treatment. The present study also demonstrates the significance of the appropriate time point for drug administration in SCI patients. Preemptive or initiatory medication in the spinal cord level has not been widely examined in SCI patients, because administering drug preemptively in these patients is almost impossible because of the unpredictable clinical occurrence of chronic pain [31]. However, it might be important to detect situations where the possibility for the development of below-level neuropathic pain is high, and the ability to alter this situation would be of considerable clinical value. Although predicting which patients suffering from SCI will go on to develop chronic central neuropathic pain is impossible, our results demonstrate that a critical time window exists in which early treatment with DBV would be effective. Recently, Tan *et al.* suggested that inhibition of early neuroimmune events could have a critical impact on the induction of long-term pain phenomena after SCI [32]. Marchand *et al.* also demonstrated that early treatment with etanercept, a tumor-necrosis-factor inhibitor, caused the reduction of mechanical allodynia after SCI, whereas delayed treatment of etanercept had no significant effect [27]. These results are consistent with the time-dependent effect of DBV observed in our present study. Collectively, these findings, including the present results, imply that repetitive DBV acupuncture therapy during the induction phase is able to produce a powerful analgesic effect on chronic central neuropathic pain and suggest the clinical use of repetitive DBV treatment as a potential novel strategy in the early management of SCI-induced neuropathic pain.

Moreover, the findings of this study demonstrate that the suppression of glial cell activation in ipsilateral, but not contralateral, spinal cord dorsal horn is closely related to the anti-allodynic and anti-hyperalgesic effects of repetitive DBV treatment during the induction phase in SCI rats. Glial cells, in particular astrocytes and microglia, have been known as important modulators or key factors of nociception. Although glia have been traditionally recognized to have simple functions that are necessary for neuronal communication in normal conditions, they are now recognized as key modulators of plasticity changes in pathophysiological conditions. Furthermore, glia can interact directly with neurons, and then, they also play important neuromodulatory and/or neuroimmune roles in the CNS [33,34]. Recently, several studies demonstrated the involvement of glia activation in chronic pain conditions, including inflammatory pain, peripheral and central neuropathic pain [20,21,23,35]. Direct metabolic inhibitors of glia activation, like minocycline and propentofylline, have been shown to have an anti-nociceptive effect in SCI rats [25,26,27,32]. Moreover, the blockade of astrocyte gap junctions by the intrathecal injection of carbenoxolone during the induction period of SCI-induced neuropathic pain reduced the development of below-level mechanical allodynia and thermal hyperalgesia and suppressed astrocytic activation in spinal cord [36]. Thus, unsurprisingly, astrocyte activation may contribute to the induction of central neuropathic pain in SCI rats. DBV stimulation of the ST36 acupuncture point also suppressed the activation of spinal cord astrocytes and reduced nociceptive behaviors in the mouse formalin test [3]. Our results showed that GFAP and Iba-1 expression in the ipsilateral lumbar 4 (L4) to L6 segments significantly increased 14 days after SCI in vehicle-treated SCI rats. However, interestingly, the increase in GFAP and Iba-1 expression was significantly decreased by repetitive DBV treatment during the induction phase. In the contralateral L4 to L6 segments, GFAP and Iba-1 expression in vehicle-treated SCI rats did not differ from that in normal animals, and repetitive DBV treatment during the induction phase did not modify GFAP and Iba-1 expression in contralateral dorsal horn examined in the present study. This result indicates that glia activation in the ipsilateral lumbar spinal dorsal horn could be caused by the damage in a spinal cord injured segment (T13) and that the mechanism underlying this remote activation of glia could ultimately lead to the development of below-level neuropathic pain. Thus, these findings suggest that the early activation of astrocytes and microglia can initiate the induction of below-level neuropathic pain.

Finally, the BBB open field locomotor test was used to examine the functional recovery by repetitive DBV acupuncture point treatment during the induction phase in SCI rats. Because the ipsilateral hindlimb was operated on in the hemisection model, we only recorded the motility of the ipsilateral hindlimb based on the locomotor rating set by Basso *et al.* [37]. Repetitive DBV treatment during the induction phase evoked a significant and rapid recovery of motor function. Thus, early repetitive DBV treatment presents the advantage of motor function recovery, because DBV-treated rats appeared to present facilitated motor functional recovery from Day 3 after SCI. Significant functional recovery was observed after repetitive DBV treatment during the induction period; however, hindlimb deficits in the saline control group were relatively prolonged for 28 days. The demyelination of axons in the injured spinal cord is a known cause of motor function deficits, and remyelination or regeneration by natural formation are extremely limited due to glial scarring and growth inhibitors contained in the environment [38]. Glia scarring is the prominent factor that inhibits axonal regeneration in the central nervous system. By limiting the formation of astrocyte scars, we can facilitate axonal regeneration physically and biochemically [39]. Another possibility is the rerouting or plasticity of injured spinal cord. Iwashita *et al.* reported that a partial recovery is available due to a rerouting mechanism in untreated SCI animals [40]. Moreover, neuroplastic changes of the CNS in response to injury have been shown to be highly susceptible to intervention during the post-injury phase [41]. The observed functional recovery here might have also been partially evoked by the reintroduction of afferent feedback signals into the injured spinal cord by the rerouted nerve. One report indicated that animals under tactile stimulation, such as direct mechanical disturbance or electrical stimulation, resulted in greater locomotion restoration [42]. Therefore, the DBV-induced constant chemical stimulation at an acupuncture point may have facilitated locomotor function recovery. Collectively, we suggest that the repetitive DBV treatment during the induction phase can facilitate motor function recovery by enhancing sensory stimulation and by suppressing secondary injury development. 

In conclusion, the present study demonstrates that repetitive DBV acupuncture therapy in spinal cord-injured rats can reduce the development of below-level mechanical allodynia and thermal hyperalgesia and can prevent glia activation in the ipsilateral spinal cord dorsal horn. In contrast, DBV treatment during the maintenance period after SCI did not modify glia expression in the spinal dorsal horn, nor below-level mechanical allodynia and thermal hyperalgesia previously established following SCI. In addition, the facilitation of motor function recovery occurred by repetitive DBV treatment. These results suggest that the repetitive application of DBV acupuncture therapy suppressed SCI-induced central neuropathic pain syndrome development and might be a potential clinical therapy for the management of SCI.

## 4. Materials and Methods

### 4.1. Animals

All experiments were performed on Sprague–Dawley male rats weighing 180 to 200 g. Animals were obtained from Orient Bio (Sungnam, Korea). The rats were housed in cages with free access to food and water. For 1 week before the study, they were also maintained in temperature- and light-controlled rooms (24 ± 2 °C, 12/12 h light/dark cycle with lights on at 07:00 h). All experimental procedures used in this study were reviewed and approved by the Animal Care and Use Committee at Korea Institute of Oriental Medicine and performed as in the NIH guidelines (NIH Publication No. 86–23, revised 1985). We made an effort to minimize animal distress and to reduce the number of animals used in this study.

### 4.2. Spinal Cord Hemisection Surgery

Spinal cord hemisection surgery was performed according to the method described by Christensen *et al.* [13]. Briefly, rats were transiently anesthetized with a combination of 2.5 mg of Zoletil 50 (Virbac Laboratories) and 0.47 mg of Rompun (Bayer Korea) in saline to reduce handling-induced stress and then mounted on the surgical field. Then, the dorsal surface was palpated to locate the cranial borders of the sacrum and the spinous processes of the lower thoracic and lumbar vertebrae. The thoracic 11 to 12 (T11 to T12) vertebrae were recognized by counting spinous processes from the sacrum. A laminectomy was performed between the T11 to T12 vertebral segments, and the lumbar enlargement region was identified with the accompanying dorsal vessel; then, the spinal cord was hemisected directly cranial to the lumbar 1 (L1) dorsal root entry zone with a No. 15 scalpel blade. We tried not to damage the major dorsal vessel or its vascular branches. All surgical procedures were performed under visual guidance using an operation microscope. Then, the musculature and the fascia were sutured, and the skin was finally apposed. After the hemisected animals recovered in a temperature-controlled incubation chamber, they were housed individually in a cage with a thick layer of sawdust and were monitored.

### 4.3. Bee Venom Treatment and Experimental Groups

First, whole bee venom (Sigma, St. Louis, MO, USA; 0.25 mg/kg) was dissolved in a 50-µL volume of saline. The apposed solution was subcutaneously administered into the Joksamli (ST36) acupuncture point on the same side as the SCI surgery (ipsilateral side). The Joksamli point was located 5 mm below and lateral to the anterior tubercle of the tibia. Previously, we reported that this dose was effective in producing anti-nociception when injected into an acupuncture point, and thus, we chose the dose for evaluating the possible anti-nociceptive effects of peripheral injection [9]. Repetitive DBV or saline injections during the induction phase were initiated on the first day post-SCI surgery and were then applied twice a day (at 8 a.m. and 8 p.m., respectively) for 5 consecutive days. During the maintenance phase, repetitive DBV or saline was administered to SCI rats from Days 15 to 20 after surgery. Although previous data suggest that repetitive DBV injection does not induce pathological changes at the site of injection [8], we examined all animals receiving DBV injections into the ST 36 acupuncture point for the appearance of edema and possible infection. In addition, we massaged the injection site area daily immediately after DBV treatment in order to prevent the accumulation of DBV in the tissues. 

### 4.4. Mechanical Allodynia Test

All behavioral assessments were performed under the ethical guidelines set forth by the International Association for the Study of Pain (IASP). Pain behavior assessments were performed one day before hemisection surgery to obtain baseline values of withdrawal responses to mechanical and heat stimuli. Then, rats were assigned randomly to each treatment group, and behavioral testing was subsequently performed blindly. During the experimental period, all behavioral tests were performed at the following time points after surgery: 3, 5, 7, 10, 14, 21 and 28 days. These tests were conducted at the same time of the day to reduce errors in relation to diurnal rhythm. Animals were placed on a metal mesh grid under a plastic chamber, and the tactile threshold was measured by applying a von Frey filament (North Coast Medical) to the mid-plantar surface of the hind paw until a positive response for withdrawal behavior was elicited. Nine calibrated fine von Frey filaments (0.40, 0.70, 1.20, 2.00, 3.63, 5.50, 8.50, 15.1 and 21.0 g) were used. They were presented serially to the hind paw in ascending order of strength with sufficient force to evoke slight bending against the paw. A brisk paw withdrawal response was considered as a positive response, for which the next filament was tested. If there was no response, the next filament was the next greater force. When animals did not respond at 21 g of pressure, the animal was recognized as being at the cut-off value. The 50% withdrawal response threshold was determined using the up-down method.

### 4.5. Thermal Hyperalgesia Test

To determine nociceptive responses to heat stimuli, paw withdrawal response latency (WRL) was measured using a previously described procedure [43]. Briefly, animals were placed in a plastic chamber (15 cm in diameter and 20 cm in length) on a glass floor and allowed to acclimatize for 10 min before thermal hyperalgesia testing. A radiant heat source was positioned under the glass floor beneath each hind paw, and paw withdrawal latency was measured to the nearest 0.1 s using a plantar analgesia meter (IITC Life Science Inc., Woodland Hills, CA, USA). The intensity of the light source was calibrated to produce a paw withdrawal response between 10 and 12 s in naive animals. The test was examined twice on both the ipsilateral and contralateral hind paws, and the mean withdrawal latency in each hind paw was calculated. The cutoff time was set at 20 s.

### 4.6. Motor Function Recovery

After the rats underwent spinal cord hemisection surgery, they were tested for motor function or coordination in an open-field test space using the BBB locomotor rating scale [36]. Briefly, the BBB scale ranges from 0 (no discernible hindlimb movement) to 21 (normal movement, including coordinated gait with parallel paw placement of the hindlimb and consistent trunk stability). Scores from 0 to 7 showed the recovery of isolated movements in the three joints (hip, knee and ankle). Scores from 8 to 13 indicated the intermediate recovery phase showing stepping, paw placement and forelimb-hindlimb coordination. In addition, scores from 14 to 21 mainly showed the late phase of recovery with toe clearance during every step phase. Only the scores of the ipsilateral hind limb on the hemisected side were examined, because there was no significant difference in locomotor function of the contralateral hind limb.

### 4.7. Western Blot Assay

All procedures for the Western blot assay were followed as described in our previous report [44]. After the mice were anesthetized by injecting a combination of 2.5 mg of Zoletil 50 with 0.47 mg of Rompun in saline, the spinal cord was obtained using the pressure expulsion method into a cooled saline-filled glass dish and was frozen quickly in liquid nitrogen. To investigate the functional changes of the L4-6 spinal cord segments, we verified the attachment site of spinal nerves in anesthetized rats. In addition, the extracted spinal segments were divided into ipsilateral and contralateral halves under a neurosurgical microscope. Subsequently, the ipsilateral and contralateral spinal dorsal horns were used for Western blot analysis. The spinal cords were homogenized with RIPA buffer (cell signaling, Beverly, MA, USA) containing protease inhibitor, phosphatase inhibitor and 0.1% SDS (sodium dodecyl sulfate). In addition, insoluble materials were removed by centrifugation at 12,000 *g* for 20 min at 4 °C. The sample protein concentrations were determined using Bradford reagents (Bio-Rad Laboratories, Hercules, CA, USA), and spinal cord lysates were separated by 10% or 15% SDS-PAGE (SDS-polyacrylamide gel electrophoresis). Subsequently, lysates were transferred to a nitrocellulose membrane. Non-specific binding was pre-blocked with 5% non-fat milk (Becton, Dickinson & Company, Franklin Lakes, NY, USA) in T-TBS and 8% bovine serum albumin (MP Biomedical) for 30 min at room temperature. Then, the membrane was incubated overnight at 4 °C with mouse anti-β-actin (1:1000, Sigma, St. Louis, MO, USA), mouse anti-GFAP antibody (1:1000, Millipore, Billerica, MA, USA) or rabbit anti-Iba1 antibody (1:1000, Abcam, Cambridge, UK) in 5% non-fat milk solution. The membrane was washed three times with T-TBS for 10 min each time and incubated with goat anti-mouse IgG horseradish peroxidase (1:5000; Calbiochem, Darmstadt, Germany) or goat anti-rabbit IgG horseradish peroxidase (1:5000; Calbiochem, Darmstadt, Germany) for 1 h at room temperature. After the membrane was washed three times with T-TBS, antibody reactive expressions were visualized using a chemiluminescence assay kit (Pharmacia-Amersham, Freiburg, Germany). The intensity of protein bands was analyzed by Image J software (Graph Pad Software, Stapleton, NY, USA, 2010).

### 4.8. Statistical Analysis

All data were expressed as the mean ± standard error of the mean (SEM) and analyzed statistically using the Prism 5.0 program (Graph Pad Software). Data from behavior studies were tested using two-way analysis of variance (ANOVA) in order to determine the significant effect of the repetitive DBV treatment. Bonferroni’s multiple comparison test as *post hoc* analysis was also performed to determine the *p-*value among experimental groups. For Western blotting analysis, column analysis was examined by Student’s *t*-test for comparisons between two mean values. *p* < 0.05 was considered statistically significant.
